# Development of an Algorithm for an Automatic Determination of the Soil Field Capacity Using of a Portable Weighing Lysimeter

**DOI:** 10.3390/s21217203

**Published:** 2021-10-29

**Authors:** Manuel Soler-Méndez, Dolores Parras-Burgos, Adrián Cisterne-López, Estefanía Mas-Espinosa, Diego S. Intrigliolo, José Miguel Molina-Martínez

**Affiliations:** 1 Agromotic Engineering and the Sea Research Group, Universidad Politécnica de Cartagena, 30202 Cartagena, Spain; manuel.ia@agrosolmen.es (M.S.-M.); adriancisterne@gmail.com (A.C.-L.); masespinosa.estefania@gmail.com (E.M.-E.); josem.molina@upct.es (J.M.M.-M.); 2 Department of Structures, Construction and Graphic Expression, Universidad Politécnica de Cartagena, 30202 Cartagena, Spain; 3 Ecology Department, (CSIC-UV-GVA), Desertification Research Center (CIDE), Carretera CV-315, km 10.7, 46113 Moncada, Spain; diego.intrigliolo@csic.es

**Keywords:** soil field capacity, irrigation automation, weighing lysimetry, irrigation technology, drainage control

## Abstract

The challenge today is to optimize agriculture water consumption and minimize leaching of pollutants in agro-ecosystems in order to ensure a sustainable agriculture. The use of different technologies and the adoption of different irrigation strategies can facilitate efficient fertigation management. In this respect, the determination of soil field capacity point is of utmost importance. The use of a portable weighing lysimeter allows an accurate quantification of crop water consumption and water leaching, as well as the detection of soil field capacity point. In this work, a novel algorithm is developed to obtain the soil field capacity point, in order to give autonomy and objectivity to efficient irrigation management using a portable weighing lysimeter. The development was tested in field grown horticultural crops and proved to be useful for optimizing irrigation management.

## 1. Introduction

Using water for farming is an essential production factor. Water availability in semiarid areas like the Mediterranean Basin is scarce and its quality is becoming increasingly worse. Faced with this challenge, high farm yields of optimum quality can be obtained by a suitable irrigation water management [1]. This implies that efficient water resources management is a main tool to ensure farms stability and sustainability [2].

Irrigation management depends on many factors which are mostly environmental and edaphic but also related to the irrigation system and plant material. In fruit crops different irrigation management strategies can be employed, e.g., partial root-zone drying or regulated deficit irrigation. However, in horticultural crops, these strategies are not feasible when water is scarce as it was summarized in a recent review [3] where other strategies are proposed mainly based on the use Information and Communication Technologies (ICTs) for efficiently managing water resources [4].

Proper efficient irrigation is very important for saving water and reducing the risk of polluting groundwater bodies mainly by nitrates percolation below the plant root-zone [3]. Managing this leaching is very complex because nitrogen is a nutrient that is closely related to horticultural crop yields and quality; the key lies in controlling the drainage of the irrigation systems [3]. This management becomes slightly more complicated if spatial edaphic and environmental variability is considered.

Different fertigation management approaches combined with available irrigation technologies can be used to manage water resources appropriately [3]. Using low-flow emitters with high irrigation frequencies helps to control the leaching rate [5]. Irrigation technologies, such as subsurface drip irrigation or using geotextiles to improve water distribution, can be used to optimize on-farm water management [6]. However, when these technologies are not capable of lowering water supply or limiting nutrient leaching by themselves, they also require intelligent irrigation scheduling [3,7].

Employing ICTs for farming is based on: (i) using sensors measuring different agri-environmental variables; (ii) dataloggers connected to sensors; (iii) servers that store data and manage information [8]. The aim is to utilize the obtained information to estimate crop water requirements by any of the available methods estimating the water balance [9,10,11,12].

Agro-climatic variables related to the soil–plant–atmosphere continuum, which can be measured by sensors and help to determine crop water requirements. The main technologies currently available for this purpose are: (i) soil moisture sensors that quantify magnitude according to different bases (matrix stress, TDR, FDR, etc.), allow to know the soil moisture content; (ii) determining plant water uptake by different technologies (vegetation indices, energy balances or water status indicators in plants) to know the water that plants use, and their values reflect in the effect of transpiration; (iii) evaporation demand tools to estimate atmospheric water demand by models that correct reference evapotranspiration with specific crop coefficients for a local area and phenological stage, and their values reflect in the effect of evapotranspiration [3].

The most accurate tool for quantifying crop water requirements by measuring water balance components is a weighing lysimeter [4,13,14]; it can be considered another technology to know evaporation demand. The potential of employing commercial lysimeters in the future is very high for their low cost and the quality data they offer. However, it is necessary to bear in mind that the apparent density of the soil inside the lysimeter must be restored in the same way as it is outside [3].

The potential of this lysimeter tool is to continuously measure any drainage and to quantify it using load cells. To date, this characteristic makes it a unique tool on the market. The obtained and quantified drainage is qualitatively evaluated by an electric conductivity sensor that estimates the evolution of its saline composition but does not define its ionic composition. To overcome this, and until reliable and affordable selective ion sensors have been developed, portable weighing lysimeters save easily removed samples for sporadic analyses at whatever time users deem appropriate. Hence this tool is considered very useful for two main reasons: (i) a crop’s evapotranspiration consumption can be accurately quantified; (ii) nutrients to minimize the leaching of elements, such as nitrogen, to lower layers can be assessed. Therefore, soil and aquifer pollution can be reduced.

Optimal water resource management involves the use of technologies such as the above, as well as following smart management methodologies [3]. It is also important to know the threshold values for each of the quantified agro-climatic variables. Knowledge of soil moisture content or soil water matrix tension at both the soil field capacity point (SFCP) and the permanent wilting point is essential for proper irrigation management [3].

According to FAO [15], soil field capacity *“refers to the relatively constant quantity of water that saturated soil contains after 48 h of drainage. Drainage occurs by water passing through pores with a diameter of >0.05 mm. However, soil capacity can correspond to pores with a diameter that varies between 0.03 mm and 1.00 mm. The soil field capacity concept is applied only to well-structured soil where the drainage of excess water is quite quick; drainage in poorly structured soils generally continues for several weeks, and this type of very poorly structured soils rarely have a clearly defined soil field capacity. Soil field capacity is better determined in the field with saturated soil and by measuring its water content after 48 h of drainage. Soil with soil field capacity feels very moist to the touch”*.

SFCP is strongly influenced by soil texture and structure. For example, the drainage of a clay-textured soil does not behave in the same way as a sandy-textured soil where saturated soil drainage finishes well before the 48 h set out by FAO. Pachés Giner [16] offers a simpler definition: *“soil field capacity corresponds to the maximum quantity of water that soil can retain against gravity’s action”*. Zhen-tao et al. [17] compared the methods followed by different authors to estimate SFCP. They indicated that SFCP is influenced by soil texture and profile depth. These authors concluded that it is possible to set up a simplified theoretical calculation method based on a dynamic method with a 3% relative drainage rate.

With sandy-texture soils, Zotarelli et al. [18] using soil moisture sensors, verified that there is a rapid water loss after irrigation. This rapid water loss is equivalent to macropore drainage. After that, the rate of soil water loss decreases, showing a clear turning point in soil moisture readings. These authors indicate that it is possible to obtain graphically the SFCP. It is also possible to detect when drainage stops by means of the herein proposed portable weighing lysimeter-based calculation method. A portable weighing lysimeter includes a drainage tank (DT) that quantifies its evolution. Thus, the time in which variation in DT weight approaches zero can be detected.

For irrigation scheduling, particularly under high frequency of water applications, it is of crucial importance to precisely detect the SFCP because it will help to determine the irrigation amount to apply on each irrigation event to reach the SFCP [19]. In practice the SFCP is used to determine the “refill point” and it is the level of soil moisture that required to be reached, but not overpassed, to continuously satisfy the crop water needs.

The objective of this work is to derive an algorithm using a weighing lysimeter that allows the SFCP to be detected and to use this information to accomplish more efficient irrigation management. This is possible thanks to the new opportunities arising from ICT allowing to use sensors in agriculture together with data interpretation algorithms for a more efficient fertigation management.

## 2. Materials and Methods

### 2.1. Test Preparation

The objective is to find an algorithm to automatically detect the SFCP. The data obtained to develop the algorithm were acquired from an experiment performed on a commercial farm from municipal region of Lorca (Murcia, Spain). The crop employed for this outdoor experiment was “*Burgundy broccoli asparagus*”, whose crop cycle lasted from 1 December 2020 to 15 March 2021. Plastic mulch was employed and rows were separated by 90 cm, with two rows of plants per drill and a 35 cm separation between plants. The irrigation system was localized and soil texture was clayey.

The dimensions of the employed portable weighing lysimeter for the cultivation tank (CT) were: 145 cm long × 65 cm wide × 50 cm deep. The CT stood on four load cells (500 kg) and resolution was 0.11 mm (100 g). The DT was placed beneath the CT and behind an intermediate control tank, quantified by means of one load cell (15 kg) with a resolution of 0.03 mm (3 g). The effectiveness for the quantification of the input/output water volumes to the soil in the CT was validated in a previous study [4].

**Figure 1:**
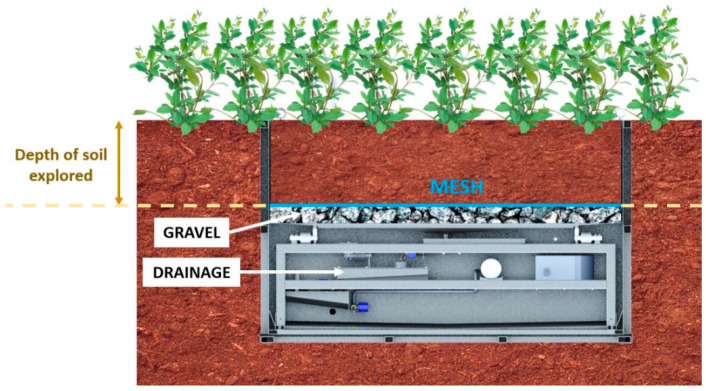
Diagram of reproducing soil inside a weighing lysimeter.

Figure 1 is a diagram of how the portable weighing lysimeter was set up. A layer of highly porous material (gravel) that does not retain water was placed on the bottom. On top of this, a sheet of draining mesh, which did not retain water. A replica of the outer soil was placed on top, with the following characteristics: (i) the outer soil was ploughed; (ii) the outer soil was at the bottom nutritional intake estimated by the farmer; (iii) the materials were added in 10 cm layers at the same relative depth as in the outer soil.

Monitoring was performed with seven plants that were planted in the soil inside the CT.

The whole portable weighing lysimeter system is protected by a cavity formed by a perimeter structure to avoid horizontal and vertical water movements from the outdoor soil to the portable weighing lysimeter interior [4]. Horizontal water movements from inside the portable weighing lysimeter to the outdoor soil are also avoided. However, downward vertical movement is facilitated to control drainage. The data obtained during the experiment were the CT weight (expressed as kg) and the DT weight (expressed as g).

### 2.2. Establishing an Algorithm to Detect SFCP by Weighing Lysimetry

According to the definition of Pachés Giner [16], when soil reaches its SFCP, it is capable of retaining all the water and, therefore, drainage stops. This study resorts to a powerful tool that quantifies drainage in real time by being able to detect the precise moment when the SFCP of the soil contained in the CT is reached. This detected time is considered the time from which variation in the DT weight approaches zero. It is lower than a given value during a time interval.

Although farmers control irrigation, some drying phases are forced after analyzing portable weighing lysimeter data to suitably detect the SFCP at certain times. Among others, the lysimeter offers these two variables:

- **Variable 1:** the CT weight, expressed as kg and determined by the following sensor structure: four load cells (500 kg) connected in parallel by a junction box linked with a six-thread cable, plus a mesh, to a 24-bit weight indicator. This indicator records the obtained data in real time with filters configured for signal peaks that do not correspond to correct readings. By means of an RS485A serial connection and the MODBUS RTU protocol, every 3 s a CAMPBELL CR300^®^ datalogger reads the record obtained by the weight indicator for the connected junction box and offers a table with the minute average of the obtained readings.
- **Variable 2:** the DT weight, expressed as g and determined by the following sensor structure: one load cell (15 kg) connected with a six-thread cable, plus a mesh, to a 24-bit weight indicator. This indicator records the obtained data in real time with filters configured for signal peaks that do not correspond to correct readings. By means of an RS485 serial connection and the MODBUS RTU protocol, every 3 s a CAMPBELL CR300^®^ datalogger reads the record obtained by the weight indicator for the connected load cell and offers a table with the minute average of the obtained readings.

As the capacity that the DT can hold is limited, the portable weighing lysimeter is equipped with a series of valves and has a programming algorithm capable of emptying this tank without losing the quantification the overall drainage. This means that the evolution of Variable 2 (DT) shows marked reductions owing to this tank being emptied. Therefore, a new variable was created that allows accumulated drainage to be quantified:

- **Variable 3:** accumulated drainage (ΣD), expressed as g, determined by the equation below:

If (DT_i_–DT_i−1_) < −1, then ΣD_i_ = ΣD_i−1_ If not, then ΣD_i_ = (DT_i_−DT_i−1_) + ΣD_i−1_ (1)

When the soil is draining, the DT progressively increases its weight until it reaches its maximum capacity. At this point, drainage is stopped and the DT is emptied. To detect the decrease in weight of the DT due to emptying, a decrease of more than one gram at two consecutive points (one minute) is discriminated. If this condition occurs, then the accumulated drainage is considered to be constant from the previous instant, until this condition is no longer fulfilled. If this condition is not fulfilled, then the variations that occur in the weight of the DT progressively increase the value of the accumulated drainage.

Once this magnitude has been analyzed, it is necessary to detect the moment when drainage ceases, in order to establish the SFCP. Furthermore, it is necessary to detect the moment when a new drainage starts in order to start a new iteration of calculations. To meet this objective, the following variables and constants were defined:

- **Variable 4:** variation in accumulated drainage (ΔΣD), expressed as g/min, determined by the algorithm below:

ΔΣD_i_ = ΣD_i_ − ΣD_i−1_ (2)

The difference in the accumulated drainage between two consecutive readings is analyzed.

○ **Constant 1:** minimum time to obtain a variation in drainage below the threshold limit (t), expressed as minutes, to set a time range to check that the variation in the DT does not exceed the set value and to consider the DT weight to be constant.

t = 30 min(3)

○ **Constant 2:** threshold limit of variation in weight over a given time period t (d), expressed as g, by establishing a maximum value for variation in the DT within a time interval t below which drainage is taken to be null and DT = ct and, therefore, SFCP has been found. This value is established to be equal to the DT’s balance resolution.

d = 3 g(4)

- **Variable 5:** accumulated drainage variation average within time interval t (ΔDt), expressed as g/min, calculated with the following equation:

ΔD_t_ = average (ΔΣD_i_:ΔΣD_i−t_) = average (ΔΣD_i_:ΔΣD_i−30_) (5)

This variable analyzes variations in accumulated drainage in the last 30 min (time t), calculates the average and is displaced over time.

The condition established to find the point at which the soil can be considered to have stopped draining is that the variation of the accumulated drainage does not exceed the value for the constant d over time t. This means that, during time t (30 min), the weight of the DT has increased below d (3 g, which is the resolution of the load cell).

○ **Constant 3:** condition for setting the SFCP (Cd), expressed as grams per minute, determined by the following equation:

Cd = d/t = 3 g/30 min = 0.1 g/min(6)

Thus, each value of ΔD_i_ must not exceed Cd to ensure that the soil is at SFCP:

ΔD_i_ < Cd ≥ ΔD_i_ < 0.1 g/min (7)

Once this condition is maintained for 5 min, the soil is considered to have reached SFCP: the SFCP value will take the value of the CT at that instant.

In order to restart the iteration and continue the search for SFCP, a draining process must be detected: the DT weight must have increased over a period of time. The following constants and conditions are established:

○ **Constant 4:** time during which an increase in weight in the DT (T), expressed in minutes, must be detected, which will allow to detect that a drainage event has occurred in the soil contained in the CT:

T = 60 min(8)

The condition to detect if a drainage event occurs is that the DT, or accumulated drainage (that is, its equivalent) increases the resolution value for at least 5 min, that is d, to obtain the following constant:

○ **Constant 5:** start condition for the iteration of new SFCP calculations as:

Ci = d/5 min = 3 g/5 min = 0.6 g/min(9)

With all these considerations, the following variable is defined:

- **Variable 6:** soil field capacity point (SFCP), expressed as kg, which shall be a fixed quantity at the moment when the above conditions are met. Its value shall be the weight of the TC at that instant; thereafter it shall remain constant until a new SFCP is obtained again.

In this way, the calculation iteration with which the SFCP will be estimated is shown.

If (ΣD_i_ – ΣD_i−1_) > 0.6 g/min during T = 60 min, this is:
If (ΣD_i_ – ΣD_i−T_) > 0.6 × T, or, in other words, 
If (ΣD_i_ – ΣD_i-60_) > 0.6 × 60 = 36 g
Then, the iteration of the drain stop search is started.
If ΔD_i_ < Cd ≥ ΔD_i_ < 0.1 g/min during t = 30 min, this is
If ΣΔD_i (1 a t)_ < 0.1 × t, or, in other words, 
If ΣΔD_i (1 a t)_ < 0.1 × 30 = 3 g
Then, CC_i_ = RC_i_ (10)

**Figure 2:**
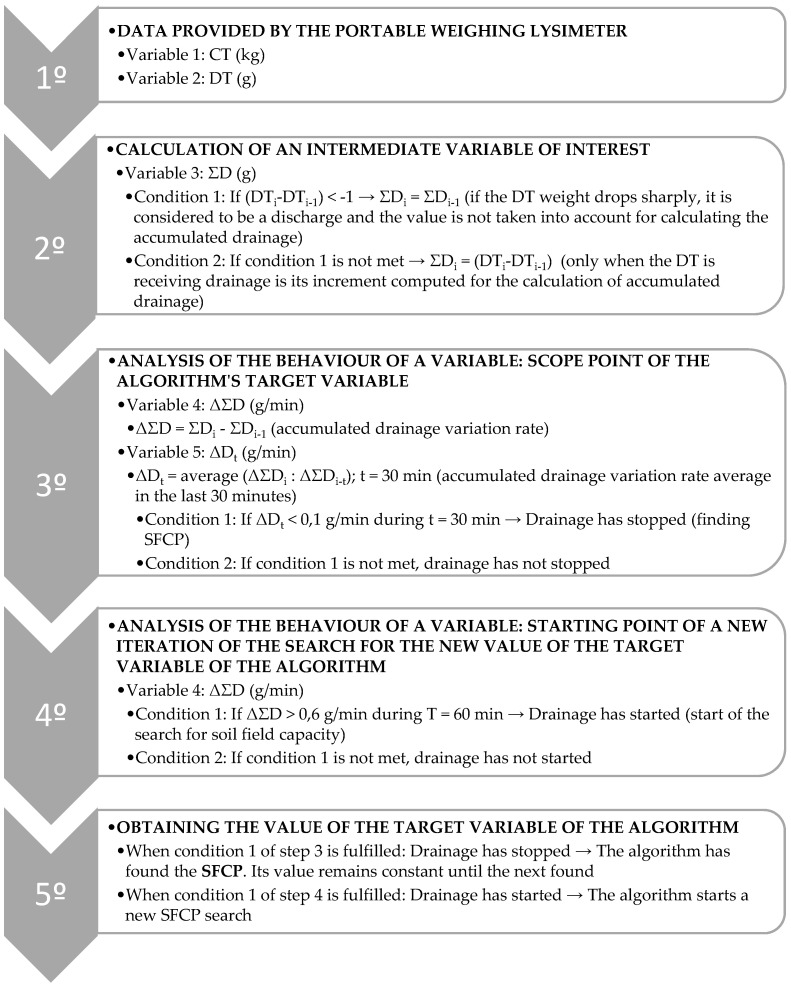
Diagram of algorithm.

The following Figure 2 shows schematically the development of this algorithm.

### 2.3. Validating Excess Irrigation Events, Drying and Soil Moisture Normalization

When a soil-drying period ends after intensive irrigation always leaves soil saturated, once again an irrigation valve is opened. Between 6 and 9 January, a large amount of water from the soil entered the CT, and the water supply to the lysimeter stopped. This was when the evolution of Variables 1 and 2 was analyzed.

On 23 January, the farmer once again irrigated. This situation continued until 18 February when irrigation stopped once more until 1 March. The evolution of Variables 1 and 2 was analyzed.

From this point, the following hypotheses were checked:

(a) Excess irrigation periods:

a Variable 1: the CT weight must be maintained at high levels, and shows not only incremented points that correspond to irrigation events, but also lowering points corresponding to loss of drainage water that occurs after an irrigation event until immediately before the next irrigation event starts.
b Variable 2: the DT weight must continue to increase its maximum capacity. Then it is immediately emptied until it reaches the weight value that it has when it is empty, and the cycle starts again.

(b) Drying periods:

a Variable 1: the CT weight must display a lowering tendency, and this variable is in accordance with: (i) If drainage still continues, it will lower more quickly than if drainage stops; (ii) If it occurs in the daytime or nighttime as it lowers more quickly in the daytime due to evapotranspiration demand.
b Variable 2: the DT weight continues to increase, but less intensely so, and releases occur increasingly less with time until they become constant.

### 2.4. Validating Irrigation Events in the Lysimeter by Comparing Irrigation Counter Data

On the irrigation line passing through the portable weighing lysimeter, an input counter and an output counter were set up, and were equipped with the two pulse emitters with a pulse per liter resolution. The values given by the counters during each controlled irrigation event were compared to the variations that took place in the CT.

The emitters of both counters are recorded through a MICROCOM^®^ model TCR200^®^ datalogger. The pulses were received in real time by the equipment, integrated with a minute frequency to show the accumulated volume.

Thus, the following variables are controlled:

- **Variable 7:** irrigation volume at initial meter (*V_i_*), expressed as liters, obtained from the data provided by the data acquisition equipment used.
- **Variable 8:** irrigation volume at final meter (*V_f_*), expressed as liters, obtained from the data provided by the data acquisition equipment used.
- **Variable 9:** irrigation volume emitted to the portable weighing lysimeter in an irrigation event (*V_lis_*), expressed as liters, obtained by the following equation:

(11) $$V_{lis}=V_{f}-V_{i}$$

- **Variable 10:** mass of irrigation emitted in an irrigation event to the portable weighing lysimeter (*Ri_c_*), expressed as kilograms, obtained by the multiplication of the irrigation volume of variable 9 per the density of water (*δ*), taken as 1 kg·L^−1^.

(12) $$Ri_{c}=V_{lis}\cdot \delta$$

- **Variable 11:** mass of irrigation emitted in an irrigation event to the portable weighing lysimeter (*Ri_lis_*), expressed as kilograms, obtained by the difference between the mass at the end of irrigation (*RC_fRi_*) and the mass at the start of irrigation (*RC_iRi_*) in the CT, plus the difference between the accumulated drainage at the end of irrigation (Σ*D_fRi_*) and the accumulated drainage at the start of irrigation (Σ*D_iRi_*) in the DT, both quantities expressed as kilograms, calculated by the following equation:

(13) $$Ri_{lis}=\left(RC_{fRi}-RC_{iRi}\right)+\left(\Sigma D_{fRi}-\Sigma D_{iRi}\right)$$

The following condition must be met:

(14) $$Ri_{c}≅Ri_{lis}$$

### 2.5. Validating the Algorithm with Data from Other Experiments

The herein developed algorithm was validated using the data obtained from another experiment performed on a commercial farm in the Almería province (Andalusia, Spain). The crop employed in this experiment was greenhouse-grown pepper with a crop cycle from 1 August 2020 to 23 May 2021, with approximately 100 cm separations between rows, 1 row of plants per drill and separations of 50–60 cm between plants. The soil where this experiment took place is franc soil, whose quantitative composition includes optimum sand, lime, and clay proportions. Such soil allows excellent farming yields thanks to its relative loose structure, because it is fertile, and it also offers suitable moisture retention. The employed irrigation system was localized.

The portable weighing lysimeter dimensions for the CT were: 150 cm long × 45 cm wide × 50 cm deep. The CT stood on four load cells (300 kg) with 0.09 mm resolution (60 g). Behind an intermediate control tank, the DT was placed beneath the CT quantified by means of one cell load (15 kg) at a resolution of 0.05 mm (3 g).

Monitoring was carried out using three plants that were planted in the soil inside the CT. This soil was reproduced by placing an outdoor soil replica and filling to a depth of 45 cm, as shown in the diagram shown in Figure 1.

In this study, different random events were controlled to validate the effectiveness of the algorithm.

## 3. Results

### 3.1. Results of the Initially Developed Algorithm to Detect SFCP with a Weighing Lysimeter

**Figure 3:**
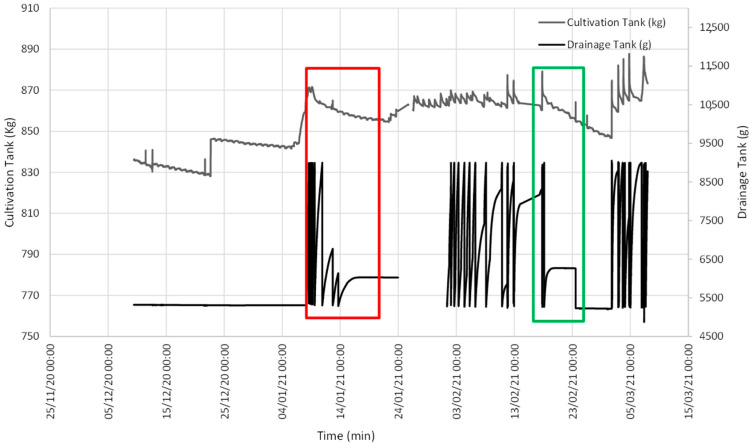
Evolution of the weight of both the CT and the DT. Red depicts the first event when SFCP was achieved. Green denotes the second event.

Figure 3 illustrates the evolution of the data obtained with the lysimeter, quantifying the evolution of both the CT and DT weight.

**Figure 4:**
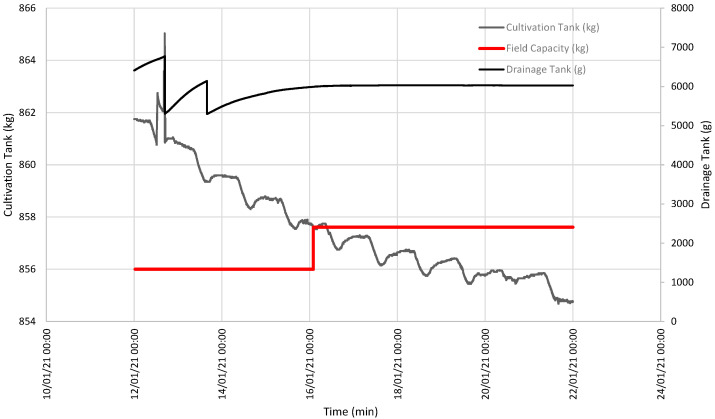
Evolution of the weight of both the CT and the DT between 12 and 22 January, and the SFCP calculation.

**Figure 5:**
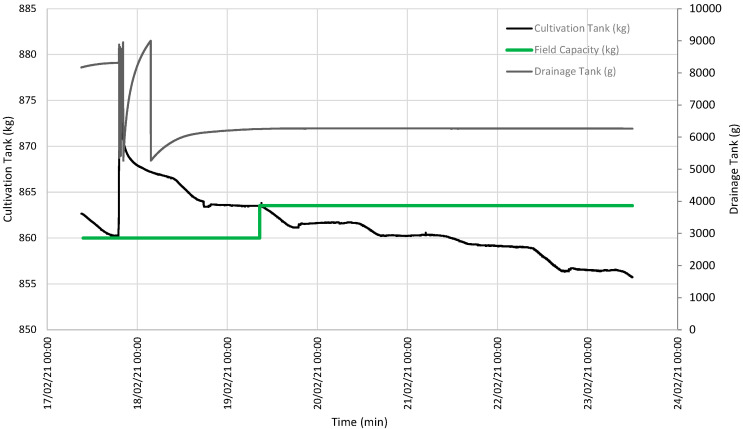
Evolution of the weight of both the CT and the DT between 17 and 24 February and the SFCP calculation.

Figure 3 illustrates two interesting episodes: one on 16 January, when soil field capacity was reached for a CT weight of 857.61 kg (Figure 4); the other episode indicated that SFCP was reached on 19 February for a CT weight of 863.52 kg (Figure 5).

**Figure 6:**
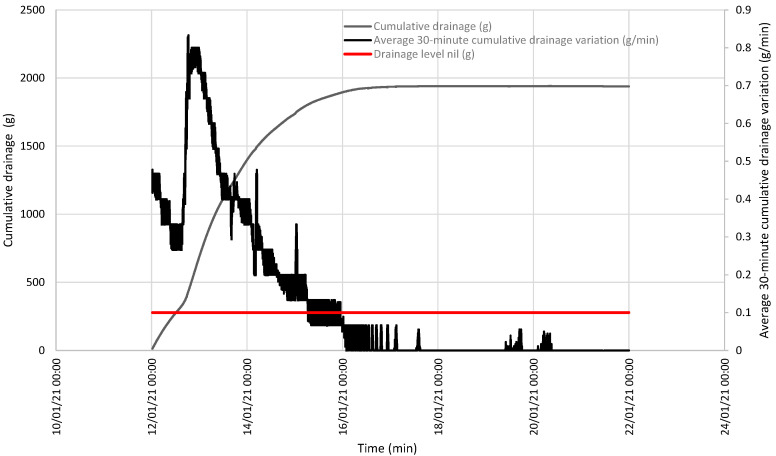
Evolution of accumulated drainage and the variation average in drainage in the last 30 min between 17 and 24 February, showing the line below which the variation average in drainage in the last 30 min is considered null.

**Figure 7:**
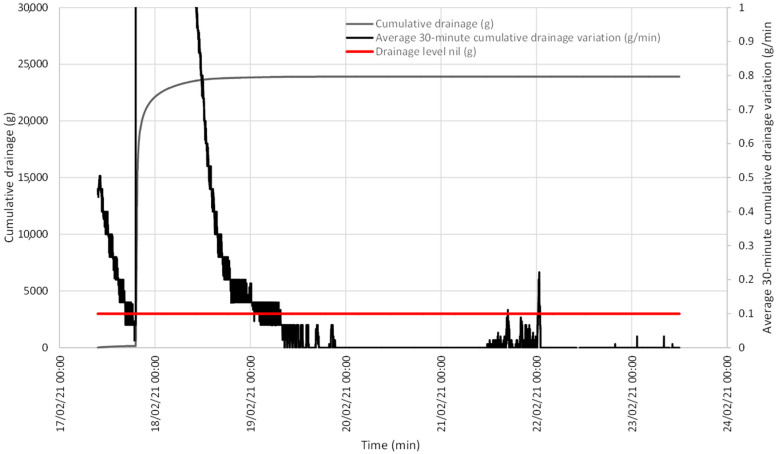
Evolution of accumulated drainage and the variation average in drainage in the last 30 min between 12 and 22 January, showing the line below which the variation average in drainage in the last 30 min is considered null.

When both events were analyzed, accumulated drainage was shown compared to the evolution of the variation average in this drainage that took place in the last 30 min. At both points, this average was below the set value of 0.1 g/min and, thus, drainage was taken to be null, as observed on the accumulated drainage curve continuity (Figure 6 and Figure 7).

### 3.2. Data about Excessive Irrigation Events, Drying and Soil Moisture Normalization

According to the graph depicted in Figure 3, on the days immediately before 6 January, the CT weight showed a downward slope, while the DT weight remained constant. This was due to water loss in soil because of evapotranspiration, with variation in the daily slope between day and night.

Between 6 and 9 January, a large amount of water from the soil inside the CT was released, and the CT weight increased to a level at which is remained relatively constant. At the same time, the DT weight brusquely went up and down owing to intense drainage. We must bear in mind that the maximum DT capacity is limited, and an internal algorithm empties it when the limit is reached to continue quantifying any drainage that takes place.

From 9 to 23 January, the downward tendency of the CT weight was once again observed with daily variations due to daytime evapotranspiration. On the first few days, the rate at which the DT weight increased slowed down as the intervals between emptying events were increasingly spaced out with time until the DT weight became constant. This meant that SFCP had been reached.

Between 23 January and 18 February, irrigation continued and exceeded the crop’s water use. The CT weight increased due to irrigation and lowered owing to evapotranspiration and drainage, but its level remained relatively constant. Except for a few days when the drainage load cell connection broke down, the DT weight frequently increased and decreased due to pronounced drainage, and its content was emptied when the maximum capacity was reached to once again receive drainage.

During the period from 18 February to 1 March, the CT input was once again limited and the same pattern as that from 9 to 23 January was followed.

### 3.3. Results of Recording Irrigation Events in the Lysimeter by Comparing Irrigation Counters Data

The irrigation that occurred on 17 February in the *Broccoli Asparagus*, Burgundy trial is analyzed by observing the initial and final meter readings. The irrigation lasted 30 min and is contrasted according to the established equations. The irrigation values counted by the meters were compared with the data recorded on the portable weighing lysimeter. This comparison shows that the quantified values are similar, and the difference is caused by the resolution of the meters (which have a pulse every liter, or kilogram of water). Table 1 shows all the data.

**Figure 8:**
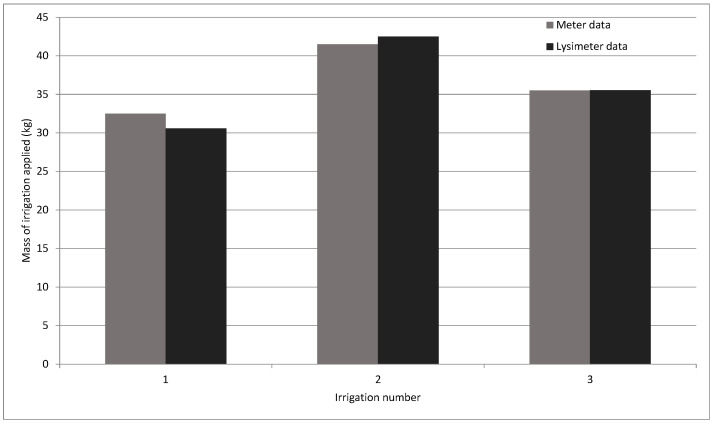
Analysis of three random irrigations by contrasting the data provided by the meters and the data provided by the portable weighing lysimeter.

To check whether this condition of similar results holds, three random irrigations are contrasted. Figure 8 shows the results of this comparison, and shows that they are always similar.

### 3.4. Results of Applying the Algorithm Using Data from Other Experiments

**Figure 9:**
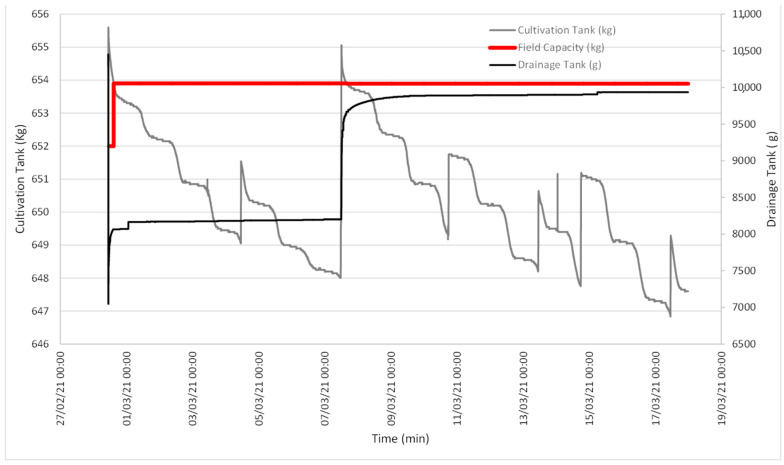
Evolution of the weight of the CT and the DT in two consecutive irrigations in the greenhouse pepper experiment, including the application of the SFCP detection algorithm developed in this work.

Figure 9 shows the evolution of the weight of the CT and the DT, applying the algorithm developed with the data from the greenhouse pepper experiment and choosing two consecutive irrigations at random. It can be seen that the SFCP is within the expected values. In both consecutive irrigations, the weight of the CT, at the moment considered as the soil has reached the SFCP, coincides in a similar value.

## 4. Discussion

Efficiently managing water resources for irrigation involves employing suitable tools by applying the correct methodology [3]. The market offers many types of technologies to monitor the agro-climatic variables of the soil–plant–atmosphere continuum [3]. The final goal is estimating the crop’s water requirements, to replace them by irrigation, and being careful to avoid salts accumulating in the soil wet areas or leaching pollutants that could potentially reach the soil and groundwater aquifers.

The tool employed in this study is a portable weighing lysimeter [4] and it is the only one of its kind on the market to date given its ability to monitor the mass of a given soil volume and, thus, its water content and, at the same time, the generated drainage mass and its qualitative characteristics (electric conductivity). One of the objectives of our research line is to seek tools that facilitate farmers’ decision for watering and fertilizing their crops. Part of this work focuses on generating algorithms that autonomously allow parameters of interest for this management to be detected.

The portable weighing lysimeter quantifies in real time not only the water interchanges taking place in the monitored soil volume, but also the fraction lost through drainage. In this way, the challenge of boosting the on-farm application of this technological innovation is presented to fulfill the objective of autonomously and objectively establishing irrigation scheduling parameters. We began by hypothesizing that when soil stops draining it is because it has reached its SFCP [12]. Then seeking to unravel when the DT increase stops allows the exact time when this SFCP has been reached to be known.

Figure 3 offers the baseline data, and two events are shown during which the DT weight became constant because SFCP had been reached. Figure 4 details the first event when SFCP was reached. SFCP took the CT weight value precisely when drainage became constant. The algorithm was also validated during the second event (represented in the graph in Figure 5), which shows that it continued to work because SFCP took the CT weight value precisely when the DT weight became stable. We can also see that the SFCP value increased during both events, as understood by the increased plant material weight and the greater water-retention capacity due to soil restructuring.

Based on portable weighing lysimeter characteristics, two excessive irrigations and drying stages were contemplated with the *Burgundy Broccoli Asparagus* experiment to hypothesize about how Variables 1 and 2 offered by the equipment would perform. When analyzing data in line with the expected hypotheses, and with both the CT and DT weights during the drying and excessive irrigation periods, the posed hypothesis could be considered valid, which indicates two facts:

1. The DT management algorithm suitably works;
2. When soil reaches SFCP, draining stops and, thus, the DT weight becomes constant.

Despite the structural design having been validated [4,8], the irrigation supply data (according to the counters set up on lateral tube) were compared to the water mass recorded by the lysimeter. The obtained results revealed that the portable weighing lysimeter well quantified the water masses supplied by irrigation.

The created algorithm was applied to the data taken from the pepper experiment and showed that the SFCP point was suitably located for the soil inside the CT; hypothetically, this soil was a replica of the outdoor soil and could, thus, help irrigation management decision making.

In order to manage irrigation, it is possible to obtain a starting point (by using a weighing lysimeter) to know the soil mass with its water content at SFCP. Thus, the reduction in CT mass is the equivalent to evapotranspiration. As it is quantified, it is possible to provide the exact amount of water for soil to recover its SFCP.

Several doubts arise about automating this process, which are also opportunities for the objectives of other research lines underway. These doubts are:

- How frequently must evapo-transpired water be reported? This depends on many factors like: water and/or energy availability; quantity, quality, and cost; crop management strategies; irrigation system characteristics, etc.
- What amount of draining should be sought? It is necessary to bear in mind irrigation water quality, supplied nutrients, absorbed nutrients, quality of the obtained drainage water, etc., to know the best wash fraction that balances crop yield loss due to environmental pollution.

To answer the question of frequency, the user should parameterize the number of daily irrigations that could be given, or how often an irrigation should be given, based on many factors. It is also totally related to the amount of irrigation that can be applied, as it will also depend on several variables. For example, it will be important to know the irrigation system, in terms of its capacity to respond to different durations of irrigation. In this sense, an irrigation system that needs a long time to discharge the water from its pipes after the solenoid valve closes will not be as manageable as one in which drainage is minimal or non-existent. On the other hand, the rainfall of the irrigation system is also of interest, in contrast to the infiltration of the soil. The slope of the land is also important in defining the relative amount of the specific weight of runoff in the total water applied to the soil.

Thus, once all the variables have been analyzed, and the working ranges for irrigation dose and frequency have been determined, the system can automate irrigation as follows:

1. Knowing the TC weight at a given moment (which will depend on the combination of parameterized dose and frequency), and knowing what the TC weight is when it is considered to be SFCP, the amount of irrigation to be applied at that moment is determined.
2. The drainage percentage that will increase the irrigation contribution will be considered.
3. From the rainfall of the irrigation system, it can be related to the amount of irrigation indicated by the portable weighing lysimeter, and the duration of irrigation to be applied is calculated.
4. The irrigation controller can be commanded to execute this instruction.

The analyzed data indicate that SFCP in absolute values does not remain constant over time but evolves according to both the soil structure and root mass development inside soil. Thus, the objective pursued with the developed algorithm is not believed to be influenced by this phenomenon because it follows the definition by Pachés Giner [16]. With other analyses, however, for which the absolute value of SFCP is relevant, knowing its evolution would be very interesting because the present work found that it does not remain constant with time.

This work contemplates starting a new research line of analyzing apparent density for different textures. To do so, analyzing the following was considered: (i) evolution of the apparent density of the soil outside the lysimeter; (ii) evolution of the apparent density of the soil in the CT of a portable weighing lysimeter; (iii) a replica of the analyses with different textures. Nevertheless, a priori the results that could be obtained were considered to help to know hydrodynamic soil performances, which were assumed to not strongly influence, (a) the theoretical basis of estimating SFCP according to the definition of Pachés Giner [16], (b) the most influential components that intervene in the quantity of evapotranspiration, such as climatic demand and a crop’s phenological status.

## 5. Conclusions

This study offered the possibility of determining in real time the CT weight in which soil is considered to be at its SFCP. With the data about CT weight, irrigation doses could be adjusted on a daily basis; that is, applying the water required to reach the CT weight that determines the SFCP. As this point can be obtained at any time, the objective of irrigation must be to generate a drainage percentage that allows salts to be washed and this SFCP value to be adjusted after each irrigation session. The development of the herein presented algorithm is taken as a starting point to accomplish autonomous irrigation scheduling and to quantify its outcomes.

## Figures and Tables

**Table 1:** Comparison of the amount of irrigation using the meters and the portable weighing lysimeter.

Description	Value	Unit
ANALYSIS OF DATA PROVIDED BY METERS		
Initial meter reading at start of irrigation	9962	m^3^
Initial meter reading at the end of irrigation	10.007	m^3^
Volume detected by the initial counter (V_i_)	0.046	m^3^
Volume detected by the initial counter (V_i_)	46	L
Final meter reading at start of irrigation	8911	m^3^
Final meter reading at the end of irrigation	8924	m^3^
Volume detected by the final counter (V_f_)	0.013	m^3^
Volume detected by the final counter (V_f_)	13	L
Irrigation volume emitted to the lysimeter (meter) (V_lis_)	33	L
Mass of irrigation emitted to the lysimeter (meter) (R_ic_)	33	kg
ANALYSIS OF LYSIMETER DATA		
Mass of the CT at the start of irrigation (RC_iRi_)	860.775	kg
Mass of the CT at the end of irrigation (RC_fRi_)	879.017	kg
Mass increase in the CT during irrigation (RC_fRi_ − RC_iRi_)	18.242	kg
Increase of accumulated drainage in the DT (ΣD_fRi_ − ΣD_iRi_)	12.346	kg
Irrigation mass quantified by the lysimeter (R_ilis_)	30.588	kg

## Data Availability

Data are contained within the article.
